# Vagotomy associated with splenectomy reduces lipid accumulation and causes kidneys histological changes in rats with hypothalamic obesity

**DOI:** 10.1590/ACB360205

**Published:** 2021-02-22

**Authors:** Kamila Aparecida Medeiros, Bruna Schumaker Siqueira, Marianela Andrea Díaz Urrutia, Elaine Manoela Porto, Sabrina Grassiolli, João Paulo de Arruda Amorim

**Affiliations:** 1 MSc. Universidade Estadual do Oeste do Paraná – Health Sciences Center – Postgraduate Program in Applied Health Sciences – Francisco Beltrão (PR), Brazil.; 2 MSc. Universidade Estadual do Oeste do Paraná – Biologics Science and Health Center – Laboratory of Endocrine and Metabolic Physiology – Cascavel (PR), Brazil.; 3 Graduate student. Universidade Estadual do Oeste do Paraná – Biologics Science and Health Center – Laboratory of Endocrine and Metabolic Physiology – Cascavel (PR), Brazil.; 4 PhD. Universidade Estadual do Oeste do Paraná – Biologics Science and Health Center – Laboratory of Tissue Biology and Reproduction – Cascavel (PR), Brazil.; 5 PhD. Universidade Estadual do Oeste do Paraná – Biologics Science and Health Center – Laboratory of Endocrine and Metabolic Physiology – Cascavel (PR), Brazil.; 6 PhD. Universidade Estadual do Oeste do Paraná – Biologics Science and Health Center – Laboratory of Tissue Biology and Reproduction – Cascavel (PR), Brazil.

**Keywords:** Monosodium Glutamate, Autonomic Nervous System, Spleen, Renal Structure

## Abstract

**Purpose:**

To evaluate the influence of autonomic vagal and splenic activities on renal histomorphometric aspects in obese rats.

**Methods:**

Thirty male Wistar rats were used, of which, 24 received subcutaneous injections of monosodium glutamate (MSG) during the first 5 days of life (4 g/kg body weight) and six control animals received injections of saline solution (CON). Five experimental groups were organized (n = 6/group): falsely-operated control (CON-FO); falsely-operated obese (MSG-FO); vagotomized obese (MSG-VAG); splenectomized obese (MSG-SPL); vagotomized and splenectomized obese (MSG-VAG-SPL).

**Results:**

The MSG-FO group animals showed a significant reduction in body weight and nasal-anal length when compared to CON-FO group animals (p < 0.05). The MSG-VAG-SPL group showed significant reduced in most biometric parameters associated with obesity. Falsely-operated obese animals showed a significant reduction in renal weight, glomerular diameters, glomerular tuff and capsule areas and Bowman’s space compared to CON-FO group animals (p < 0.05). There was a significant reduction in diameter, glomerular tuft and capsule areas, and Bowman’s space in MSG-VAG, MSG-SPL, MSG-VAG-SPL groups when compared to the MSG-FO group.

**Conclusions:**

Vagotomy associated with splenectomy induces a reduction in the adiposity and causes histological changes in the kidney of obese rats.

## Introduction

Chronic kidney disease (CKD) is one of the most important public health problems worldwide, and its main implications involve damage to nephron structures and loss of kidney function. It has a progressive character and is related to high morbidity and mortality^1^. This disease is identified by the gradual destruction of nephrons caused by increased intraglomerular pressure and hyperfiltration. The pathogenic mechanisms that influence this disease converge in a common environment, which results in progressive interstitial fibrosis, peritubular capillary loss with hypoxia and destruction of functional nephrons as a result of tubular atrophy^2^^,^ 3.

Currently, it has been considered the role of subclinical inflammation in the evolution of chronic-degenerative diseases^4^. Inflammation is identified as a physiological process, in which there is a response to different stimuli such as infections, physical, chemical and antigenic changes or traumatic damage. The inflammatory response needs to be strictly ordered, since deficiencies or excess responses are closely associated with morbidity and mortality. For example, the inflammation that started in the glomerulus due to some trauma, causes innumerable harmful mechanisms through the activation of the immune system in a continuous and accentuated way, which may affect the interstitial tubule space. In this scenario, there is evidence of activation of the immune system over early and late stages of CKD. On the other hand, studies reveal the existence of a negative relationship between circulating levels of inflammation mediators and the stage of the disease^5^.

Studies show that obesity is an independent risk factor for CKD^6^^,^ 7. Research shows that obese patients in the United States are four times more likely to develop CKD than nonobese patients. Obesity is believed to interfere with pathophysiological changes that favor kidney damage. The increase in lipids in macrophages is able to transform the phenotype of cells and benefit the appearance of a proinflammatory environment responsible for the pathophysiological changes of the kidney related to obesity. There is evidence of an association of numerous proinflammatory cytokines produced by adipose tissue and inflammatory cells with kidney damage caused by obesity^8^.

Obesity causes several structural, hemodynamic and metabolic changes in the kidneys. Most of these changes can be compensatory responses to the systemic increase in metabolic demand observed in obesity. However, in some cases, kidney damage becomes clinically affected as a result of compensatory failure. Obesity-related glomerulopathy (ORG) is the best known^9^. The literature shows an association between glomerulopathy and inflammation. It is noteworthy that glomerular diseases indicate faster renal function deterioration when compared to other CKD etiologies. Glomerular injury can be caused by several immunological mechanisms^7^. Sympathetic activity may also be associated with the evolution of renal failure. According to Luo *et al.* 10, the increase in sympathetic tone significantly alters renal function. In addition, renal inflammation is highly involved with the vagus nerve, mainly through the presence of the inflammatory reflex, wherein the afferent vagus nerve detects peripheral inflammation and the signal is transmitted through the central nervous system to the efferent vagus nerve and the spleen to relieve inflammation^11^.

Thus, the aim of the present study was to evaluate the relationship between autonomic vagal and splenic activities on renal histomorphometric aspects in MSG-obese rats.

## Methods

The experimental procedures were in accordance with the Ethical Principles in Animal Experimentation adopted by the Brazilian College of Animal Experimentation and were approved by the Ethics Committee of Animal Use at Universidade Estadual do Oeste do Paraná (protocol. 0906/2017).

Thirty male Wistar rats were used; of these, 24 received injections of monosodium glutamate (MSG) (4 g/kg body weight) during the first 5 days of life^12^. In the same period, 6 control rats (CON) received subcutaneous injections of equimolar saline. The animals were adapted and maintained at the vivarium of the Center for Biological and Health Sciences (CCBS) from Universidade Estadual do Oeste do Paraná, housed in collective polyethylene cages (43 × 30 × 15 cm), under controlled temperature, 22 ± 25 °C,12 hour-photoperiod (light period 7:00 ~ 19:00 h).

### Experimental design

Considering the induction of obesity with MSG, vagotomy and splenectomy, five experimental groups were organized (n = 6/group), as follows: falsely-operated control (CON-FO), falsely-operated obese (MSG-FO), vagotomized obese (MSG-VAG), splenectomized obese (MSG-SPL), vagotomized and splenectomized obese (MSG-VAG-SPL).

### Surgical procedures

Splenectomy: Animals in the MSG-SPL and MSG-VAG-SPL groups underwent splenectomy at 60 days of age. For the surgical procedure, the animals were intraperitoneally anesthetized, with a mixture (v:v) of xylazine (0.2 mg/g) and ketamine 0.5 (mg/g) of weight of each animal. Subsequently, the animals were laparotomized, the spleen located, the splenic vessels were tied with a 3 × 3 mm green polyester surgical thread (PolySuture), the spleen was removed and weighed, and the cut sutured with the same type of surgical thread used to tie the vessels and animals returned to the vivarium. Falsely-operated groups (CON-FO) underwent the same processes, except the removal of the spleen.

Subdiaphragmatic vagotomy: Animals in the MSG-VAG and MSG-VAG-SPL groups were subjected to subdiaphragmatic vagotomy at 60 days of age, according to the protocol by Balbo *et al*.^13^ with adaptations. For vagotomy, the animals were intraperitoneally anesthetized as previously described. Then the animals were shaved in the ventral region, performing antisepsis of the surgical field using polyvinyl pyrrolidone-iodine (PVPI), a cutaneous ventral incision of approximately 2 cm, inferior to the sternum, in the midline of the abdomen. A similar incision was made in the abdominal muscle wall. The intestine was caudally retracted and the liver cranially, to expose the esophagus. With the aid of a magnifying glass, the anterior and posterior branches of the vagus nerve, which are located near the surface of the esophagus, were gently dissected and sectioned with scissors and precision tweezers. In animals selected for false vagotomy (FO groups), after opening the peritoneal cavity, the cavity was explored, the vagus nerve was handled and detached from the esophagus, but not sectioned. At the end, the muscle incision was closed with continuous absorbable suture and the skin incision with simple non-absorbable suture.

### Euthanasia, organ weight and adiposity

At 150 days of age, the animals were weighed and euthanized by decapitation in guillotine. Animals were submitted to abdominal-pelvic laparotomy to remove organs and tissues, which were weighed on a digital scale. The weight of organs and tissues was expressed in standardized units corresponding to the gram of organ/100 g body weight (relative weight = organ or tissue weight / body weight **×** 100).

To evaluate the development of obesity in the MSG group, weights of fat deposits in the abdominal cavity (retroperitoneal, mesenteric and perigonadal) and subcutaneous (inguinal) were measured and the Lee index was calculated in all animals, using the relationship between cube root of body weight in grams (g) by nasal-anal length (cm)^14^.

### Morphological and morphometric analysis of the kidneys

Kidneys were fixed in alcohol, formaldehyde and acetic acid (ALFAC) for 24 h, washed in running water and stocked in 70% alcohol. Subsequently, they were processed for light microscopy, with embedding in Paraplast Plus (Sigma-Aldrich). For morphological analysis, semiserial cuts of 5 μm thickness were made, using a manual rotary microtome (Olympus 4060), equipped with a disposable steel razor. The sections obtained were deparaffinized with xylol, hydrated with distilled water and stained with hematoxylin and eosin (HE) for analysis.

For morphometric analysis, one kidney histological section was used and three subsequent ones were discarded along the organ, making an average of 10 sections/animal. Fifty glomeruli were selected per kidney and measured for diameter of the glomerular tuft, glomerular tuft area, capsule area and Bowman’s space. To know the area of Bowman’s space, the capsule area was calculated by subtracting the area of the glomerular tuft^15^^,^ 16. All sections were observed using an Olympus BX60 microscope. Images of renal glomerulus were observed at 400× magnification. The images were recorded using an Olympus DP71 digital camera with the DP Controller software v. 3.2.1.276 and analyzed using the Image Pro-Plus software v. 4.1. The results were expressed in micrometers (μm).

### Statistical analysis

All data were expressed as mean ± standard error, applying the analysis of variance – ANOVA, followed by Tukey’s post-hoc test. The differences were considered statistically significant when p < 0.05. Statistical analyses were performed using the Sigma Plot software (version 11.0; Systat Software Inc., San Jose, CA, USA).

## Results

### Biometric parameters associated with obesity

At the end of the experimental period (150 days of life), the animals in the MSG-FO obese group showed a significant reduction in body weight and nasal-anal length when compared to animals in the non-obese CON-FO group (p < 0.05). About the other parameters associated with obesity (Lee index and fat deposits), the MSG-FO group animals showed a significant increase in these measures when compared to CON-FO animals (p < 0.05). The MSG-VAG group not showed significant difference of body weight and nasal-anal length; however, significantly reduced the Lee index and retroperitoneal, perigonadal and mesenteric fat deposits when compared to MSG-FO group (p < 0.05). The MSG-SPL animals significantly reduced the Lee index and retroperitoneal fat when compared to MSG-FO animals (p < 0.05); however, the removal of the spleen did not affect the other fat deposits. The MSG-VAG-SPL group showed a significant reduction in most of the biometric parameters associated with obesity when compared to MSG-FO group (p < 0.05) (Table 1).

**Table 1:** Biometric parameters of the different groups at the end of the experimental period.

Parameters		CON-FO		MSG-FO		MSG-VAG		MSG-ESP		MSG-VAG-ESP
Body weight (g)		439.60 ± 6.41		294.00 ± 13.39*a		292.00 ± 5.97a		306.80 ± 10.18ab		262.20 ± 3.60d
Nasal-anal length (cm)		23.25 ± 0.12		19.30 ± 0.27*a		20.20 ± 0.30a		20.40 ± 0.24b		19.20 ± 0.20abd
Lee index		327.00 ± 1.49		343.79 ± 2.11*a		328.78 ± 3.88b		330.37 ± 2.02bc		332.82 ± 3.47bcd
Retroperitoneal fat (g/100 g BW)		1.53 ± 0.10		1.70 ± 0.26*a		0.68 ± 0.02b		0.95 ± 0.04c		0.74 ± 0.02bd
Perigonadal fat (g/100 g BW)		1.54 ± 0.07		2.5 ± 0.09*a		1.93 ± 0.09b		2.75 ± 0.05ac		1.99 ± 0.05bd
Inguinal fat (g/100 g BW)		0.28 ± 0.03		0.59 ± 0.07*a		0.84 ± 0.17b		0.57 ± 0.02ac		0.48 ± 0.10acd
Mesenteric fat (g/100 g BW)		0.99 ± 0.08		2.29 ± 0.17*a		1.53 ± 0.18b		2.13 ± 0.11bc		1.48 ± 0.19d

Values expressed as mean ± standard error. N = 6 animals/group. BW = body weight. Analysis of variance - ANOVA (one-way), followed by Tukey’s test,

* = p < 0.05 between the CON-FO and MSG-FO groups. Different lowercase letters a, b, c, d = p < 0.05, when comparing obese groups.

### Renal histomorphometric analysis

**Figure 1:**
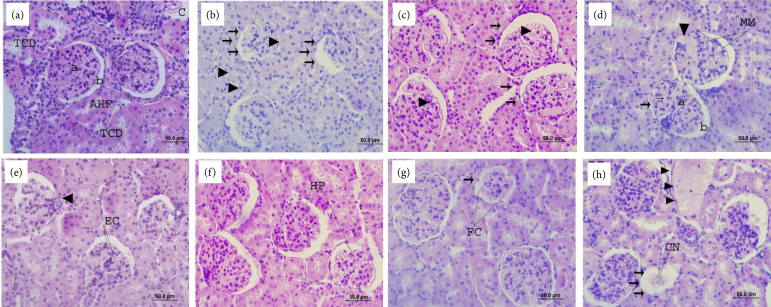
Renal glomerulus photomicrograph of the animals from different experimental groups. (a) Glomerulus with normal aspect in the animals from CON-S group, showed renal corpuscles, distal contorted tubules (DCT), segments loops of Henle (SLH), blood capillaries (*), glomerular tuft (triangle), Bowman’s space (circle); (**b**) Reduced glomerulus and with altered appearance in animals of the MSG-FO group (*arrows*), immature tubules (*arrowhead*); (**c**) Glomeruli with increased diameters (*arrows*), diffuse thickening of the capillary walls (*arrowhead*); (**d**) Increase in the mesangial matrix (MM), nodular lesions (arrowhead), dilated capillaries (*thin arrow*), capillary membranes are thickened (*thick arrow*); (**e**) Glomerular lobulation (*arrowhead*), thickening of capillary walls (EC); (**f**) Capillary wall thickening and hypercellularity (HP); **(g**) Glomerular necrosis (*arrow*) and crescent formation (CF); (**h**) Renal infarction in animals MSG-VAG-ESP (*arrow*), necrotic cells (NC), blood vessel (*arrowhead*). Staining = Harris hematoxylin and eosin.

The animals in the CON-FO group showed preserved renal structure, with a cortical region of granular appearance, containing nephrons, renal corpuscles, contorted tubules and thin segments loops of Henle (Fig. 1a). The kidneys of MSG-FO group animals showed altered histological structure, with immature glomerulus, tubules and cartilage, surrounded by loose and undifferentiated mesenchymal tissue (Fig. 1b). Some glomeruli slightly enlarged with diffuse thickening of the capillary walls (Fig. 1c). Glomerulus with a prominent increase in the mesangial matrix, forming several nodular lesions. The dilation of glomerular capillaries was evident and some basal capillary membranes are thickened (Fig. 1d). There was an accentuated glomerular lobulation with a greater number of mesangial cells and amount of mesangial matrix, and thickening of capillary walls (Fig. 1e). Capillary wall thickening and hypercellularity were also evident (Fig. 1f). Some glomeruli were found with focal glomerular necrosis and crescent formation (Fig. 1g). In the MSG-VAG and MSG-VAG-SPL animals, the kidneys were found with multiple areas of renal infarction characterized by marked pallor, extending to the subcapsular surface (Fig. 1h).

In the histomorphometric analysis of renal parameters, the MSG-FO group showed a significant reduction in renal weight and diameters of glomerular tuft area, capsule area, and Bowman space, when compared to CON-FO group (p < 0.05) (Table 2). The groups MSG-VAG, MSG-SPL, MSG-VAG-SPL showed a significant reduction in diameter and area of glomerular tuft, area of the capsule and the Bowman’s space were found when compared to the MSG-FO group, (p < 0.05) (Table 2).

**Table 2:** Glomerular histomorphometry of the different experimental groups at the end of the experimental period.

Parameters		CON-FO		MSG-FO		MSG-VAG		MSG-ESP		MSG-VAG-ESP
Renal weight (g/100 g BW)		0.30 ± 0.009		0.23 ± 0.01*a		1.88 ± 0.41b		0.23 ± 0.05ac		0.24 ± 0.01acd
Diameter of the glomerular tuft (μm)		119.98 ± 1.25		93.15 ± 0.82*a		33.83 ± 0.42b		35.19 ± 0.37bc		34.2 ± 0.37bcd
Area of the glomerular tuft (μm^2^)		9176.39 ± 106.32		8081.42 ± 124.21*a		869.10 ± 12.41b		917.96 ± 18.33bc		863.54 ± 15.25bcd
Bowman’s capsule area (μm^2^)		7517.01 ± 108.86		5620.90 ± 101.96*a		637.30 ± 11.30b		672.48 ± 15.44bc		599.52 ± 12.20bcd
Bowman’s space area (μm^2^)		119.98 ± 1.25		93.15 ± 0.82*a		33.83 ± 0.42b		35.19 ± 0.37bc		34.20 ± 0.37bcd

Values expressed as mean ± standard error. N = 6 animals/group. Analysis of variance – ANOVA (one way), followed by Tukey’s test,

* = p < 0.05 between the CON-FO and MSG-FO groups. Different lowercase letters a, b, c, d = p < 0.05, when comparing obese groups.

## Discussion

Obesity is associated with insulin resistance, diabetes, dyslipidemia and hypertension. Collectively, these conditions comprise the metabolic syndrome, which involves a low-grade chronic proinflammatory state^17^. In addition, obesity is the main cause of chronic kidney disease, especially in terminal kidney disease, with ORG being the most well-known kidney disease associated with obesity^18^^,^ 19.

In this research the animals of MSG-FO group showed a significant reduction in body weight and nasal-anal length when compared to CON-FO group. This reduction can be attributed to the side effect of the application of MSG, since the endocrine alterations includes the reduction of circulating levels of growth hormone (GH), due to the permanent destruction of neurons in the hypothalamus arcuate nucleus, GH-RH production site. According to results, despite the lower body weight and nasal-anal length of these animals, there was a significant increase in the Lee index and fat deposits. The lower secretion of GH implies in the reduction and retardation of the animal growth, due to the inadequate growth of the volume and the number of cells. This hormone is considered a calorific hormone, which produces lipolysis and anabolism and, in addition to being diabetogenic, its reduction implies the failure of fat mobilization, thus contributing to the increase in the adipose tissue of these animals^20^^–^ 23.

In MSG-VAG animals there was a significant reduction in the Lee index and in the retroperitoneal, perigonadal and mesenteric fat deposits. Souza *et al*.^24^ showed that vagotomy promotes weight loss and reduced food intake more importantly in the first postoperative days, one of the explanations for this fact is that the procedure blocks the feeding inhibitory effect, consequently, these animals ingest smaller amounts and more frequent portions of liquid diet, and larger amounts and less frequent portions of solid diet, possibly explained by the reduction in tone of the pyloric sphincter. in addition, vagal activities are associated with gastric emptying^25^^,^ 26. It is known that the digestive tract loses control from the central nervous system after vagotomy^27^, what probably happened the animals in this research.

King *et al*.^28^ evidenced that the reduction in the Lee index of vagotomized animals is due to hypophagia, consequently reducing the weight and the percentage of fat deposits. Based on these studies, it can be deduced that the reduction in the Lee index and fat deposits in these animals occur by two possible mechanisms: reduction of food intake and interruption of the vagal stimulus to the pancreas, reducing hyperinsulinemia.

In MSG-SPL animals there was a significant decrease in the Lee index and retroperitoneal fat, not affecting the other fat deposits. According to Gotoh *et al*.^29^ splenectomy decreases food intake. However, Maury and Brichard^30^ describe an increase in retroperitoneal fat, as well as an increase in the area of adipocytes. About lipid metabolism, Alberti *et al*.^31^ evidenced that animals submitted to splenectomy, showed increase of total cholesterol and LDL-cholesterol fraction concentrations. As well as the splenectomy is related to changes in the lipid metabolism that are reverted by spleen tissue implants.

Carvalho and Saad^32^ showed that there was a decrease in fat deposits and an increase in insulin sensitivity in obese and splenectomized mice, reflected by a reduction in blood glucose. There was also a reduction in the infiltration of macrophages into the liver and in the adipose tissue of splenectomized mice. According to Leite *et al*.^33^, splenectomy reduces obesity, hyperinsulinemia and insulin resistance in MSG rats, changes that may be related to a reduction in the inflammatory process originating from the spleen.

In this study, the MSG-VAG-SPL group showed a significant reduction in most of the biometric parameters associated with obesity when compared to MSG-FO animals. There are no data in the literature evaluating this double surgery. However, there is a possible relationship with the decrease in biometric parameters with the dual surgery. It is known that isolated vagotomy is already sufficient to reduce dietary intake and lead to weight loss in humans and laboratory animals that showed a decrease in daily feed intake, reducing weight and fat deposits. This effect is due to the loss of efferent vagal pathways, which arouse appetite and endocrine changes^34^.

The first research demonstrating parasympathetic innervation in the abdominal viscera is the study by Swan^35^, which showed terminations of the posterior vagal branch in the celiac plexus. The celiac plexus is responsible for the innervation of organs in the retrodiaphragmatic portion of the digestive system, in which it contributes to the innervation of the spleen^36^. As in this study, several studies suggest or demonstrate the existence of a vagus nerve-celiac ganglion connection^37^^–^ 39.

It was demonstrated that CON-FO animals showed preserved renal structure, unlike animals in the MSG-FO group, which have altered histological structure, in addition to immature glomerulus, tubules and cartilage, surrounded by loose and undifferentiated mesenchymal tissue, enlarged glomeruli with diffuse thickening of capillary walls, prominence of the mesangial matrix and focal glomerular necrosis. Researchers demonstrated that obesity causes a metabolic overload and triggers a series of changes such as arterial hypertension, diabetes mellitus and abnormal lipid metabolism, considered the main causes of chronic kidney disease^3^.

Weisinger *et al*.^40^ hypothesized the direct relationship between obesity and kidney injury, demonstrating the association between morbid obesity, proteinuria, glomerulomegaly and focal segmental glomerulosclerosis, stating that obesity has a direct relationship in the failure of renal function. Kambham *et al.* 41 associated glomerulopathy with obesity and, according to the authors, kidney damage associated with obesity does not depend on hypertensive or diabetic disease. According to Zhu and Scherer^42^, obesity causes metabolic disorders that can affect renal function, evidencing perihilar focal segmental glomerulosclerosis associated with obesity.

In the histomorphometric analysis of renal parameters, the MSG-FO group showed a decrease in renal weight and diameters of glomerular tuft area, capsule area, and Bowman’s space when compared to CON-FO group. According to Pereira *et al*.^43^, obesity raises the basal metabolic needs, increasing blood flow, cardiac output and blood pressure. The authors argue that part of the cardiac output is destined for the kidney, with vasodilation of the afferent arteriole, increased renal plasma flow and glomerular hyperfiltration.

In accordance with Lee *et al*.^44^, changes in fat deposits that accompany obesity are associated with the progression of kidney disease, due to epithelial and mesangial cell damage. Paula *et al*.^45^ also showed the progression of kidney disease and glomerular changes, such as vasodilation of the afferent arteriole with increased renal blood flow, glomerular hypertension and hyperfiltration, and thickening of the glomerular and tubular basement membranes. According to these authors, the mechanisms responsible for renal vasodilation in the obese are not well understood; however, they may be related to the feedback mechanism of the macula densa, in which the increase in sodium reabsorption in the proximal segments of the nephron leads to a reduction in the supply of sodium chloride to the distal tubule, stimulating the macula densa to cause afferent vasodilation and to secrete renin resulting in greater expansion of the extracellular volume.

According to the results of this study, there was a significant reduction in the diameter and area of glomerular tuft, capsule area and Bowman’s space in the MSG-VAG, MSG-SPL, MSG-VAG-SPL groups when compared to MSG-FO group, mainly in the MSG-VAGMSG-VAG-SPL groups. However, the MSG-VAG group showed a significant increase in renal weight. The reduction in renal parameters of operated groups can be explained by renal innervation, which originates from the renal nervous plexus, formed by sympathetic fibers from the thoracic splanchnic nerve, and parasympathetic nerves from the vagus nerve, in addition to abdominopelvic splanchnic nerve fibers, suggesting that lack of innervation results in renal atrophy^46^^,^ 47.

To date, there are no studies relating vagotomy combined with splenectomy and MSG-induced obesity model. Considering that there are significant changes in the renal parameters of MSG-VAG-SPL animals when compared to MSG-FO group, it is worth mentioning that there are studies revealing an important association between vagus autonomic activity and the functioning of the immune system, affecting mainly the spleen^48^^–^ 50. The interaction between the efferent vagus nerve and the splenic nerve can occur in the suprarenal, superior mesenteric or celiac adrenal ganglia^51^^,^ 52.

Sympathetic activity, through different mechanisms, may also be associated with the evolution of renal failure. A study shows that with stimulation of renal sympathetic fibers, there is an increase in the production and release of norepinephrine, on the other hand, when there is an interruption of sympathetic nerve stimulation, there is a reduction in its production and release. In CKD, sympathetic hyperactivity is evident in the earlier clinical phase of the disease, revealing a direct association with the severity of the state of renal failure^53^. Another study showed that patients with CKD had sympathetic renal denervation, with consequent changes in renal structure^54^.

It is known that the spleen can undergo changes due to numerous conditions, due to its wide variety of functions, including obesity. Studies show that the participation of the spleen in inflammation and obesity is evidenced by splenectomy^55^^,^ 56. The results demonstrated that splenectomy influences the renal parameters of obese animals. According to Kopple^57^ and Hall *et al*.^58^ splenectomy reduces food intake in these animals and, consequently, leads to protein restriction, causing low weight and decrease in kidney size, resulting in glomerular injury.

## Conclusion

The vagotomy associated or not with splenectomy induce a reduction in the adiposity and causes histological changes in the kidneys of obese rats.
